# Color stability of enamel treated with different antioxidant agents following at-home bleaching with 10% hydrogen peroxide

**DOI:** 10.1590/1678-7757-2024-0056

**Published:** 2024-07-08

**Authors:** Rodrigo Chiles PEREIRA, Letícia Vasconcelos Silva de SOUZA, Matheus KURY, Iago César Ribeiro Teles MATOS, Reginna Vyctória da Trindade Souza de Melo CARNEIRO, Sandrine Bittencourt BERGER, Vanessa CAVALLI

**Affiliations:** 1 Universidade Estadual de Campinas Faculdade de Odontologia de Piracicaba Departamento de Odontologia Restauradora Piracicaba SP Brasil Universidade Estadual de Campinas, Faculdade de Odontologia de Piracicaba, Departamento de Odontologia Restauradora, Piracicaba, SP, Brasil.; 2 Universidade do Norte do Paraná Faculdade de Odontologia da Londrina Departamento de Odontologia Restauradora Londrina PR Brazil Universidade do Norte do Paraná, Faculdade de Odontologia da Londrina, Departamento de Odontologia Restauradora, Londrina, PR, Brazil.

**Keywords:** Antioxidants, Tooth bleaching, Color, Hydrogen peroxide

## Abstract

**Objective:**

This study evaluated the color stability of enamel submitted to 10% hydrogen peroxide (HP) followed by antioxidants agents, and the pH and antioxidant activity (AA%) of these agents.

**Methodology:**

Bovine enamel-dentin blocks were randomly distributed into groups (n=10/group): GNC (negative control: no treatment); GPC (positive control: bleaching only); TOC_10% (HP+10% α-tocopherol); GT_10% (HP+10% green tea extract); GS_5% (HP+5% grape seed extract); SA_10% (HP+10% sodium ascorbate); QUI_10% (HP+10% quinoa extract); and QC_1% (HP+1% quercetin). Color (ΔE_00_) and whiteness index (ΔWI_D_) changes were analyzed using a digital spectrophotometer. The pH and AA% were determined using a pH meter and the DPPH method, respectively. Data were analyzed by ANOVA/Tukey’s and Dunnett’s tests (α=0.05).

**Results:**

At 14 days post-bleaching, GNC promoted the lowest ΔWI_D_ and ΔE_00_ (p<0.05), and no differences were found between GPC and the remaining groups submitted to the antioxidant agents (p>0.05). QC_1% and QUI_10% exhibited acidic pH levels (3.64 and 4.75, respectively), whereas TOC_10% and GS_5% exhibited alkaline pH (7.07 and 7.64, respectively). No differences in AA% were found between the agents (p>0.05), ranging from 92.6 to 97.6%.

**Conclusion:**

The antioxidant agents did not interfere in bleached enamel color stability, showing satisfactory antioxidant activity. However, QUI and QC gels displayed acidic pH. Clinical significance: The antioxidants evaluated showed high AA% and no impact on post-bleaching color stability, suggesting that their capacity to recover bond strength demonstrated elsewhere would not compromise the esthetic efficacy of tooth bleaching. However, those with acidic pH should be used with caution due to potential enamel damage.

## Introduction

Tooth bleaching is a widespread procedure due to its proven conservative methods and long-term effective outcomes to resolve dental discoloration.^1^ Evidence shows that tooth bleaching may impact the patient’s quality of life.^2^ This treatment consists of applying hydrogen (HP) or carbamide (CP) peroxide gels on the buccal surface of dental enamel.^3^ Traditionally, in-office bleaching is performed using high concentrations of HP or CP, whereas at-home therapy uses low-concentrated CP.^3^ More recently, low-concentrated HP has been also employed in at-home therapy due to its reduced application time compared to CP.^4^ Theoretically, HP would decompose into reactive oxygen species (ROS) and penetrate towards dental structure through the porosities of enamel prisms.^5^ In dentin, these ROS break down long-chained organic molecules, the so-called chromophores, into smaller ones, and teeth become whiter.^6^

Despite being minimally invasive, the bleaching gels might cause microstructural changes in dental enamel, such as decrease in the surface microhardness^7^ and the bond strength (BS),^8^ hindering the quality of immediate bonding procedures. Such impairment of short- and long-term adhesion of the resin to the enamel could be a consequence of the oxygen by-products’ entrapment in the dental structure and the formation of pores, preventing appropriate light-curing of composite resin-based materials.^9^ In this critical scenario, it is important to highlight that tooth bleaching is performed before restorative procedures, as the color change promoted by HP or CP agents in composite resins would not match the color of teeth, rendering changes that are not acceptable.^10^ Therefore, literature has already reported waiting times from seven^11^ to 21 days^12^between the last bleaching gel application and the restorative procedures. However, one must consider that the waiting time might not be consistent with the patient’s desires and needs.^13^

In this direction, several antioxidants have been studied over the years to reverse the immediate bleached enamel bond strength, such as sodium ascorbate (SA),^14-16,19^ α-tocopherol (TOC),^17,18,19^green tea extract (GT),^15,17,18^ grape seed extract (GS),^20^ and quercetin (QC).^19,21^ In addition, some other natural compounds show antioxidant activity, such as quinoa (QUI), which can be a clinical option to reverse bond strength.^22^A recent literature review on the use of antioxidants on bleached teeth concluded that the efficacy of natural agents to recover the immediate shear and tensile BS was consistent among the studies published over the last 20 years.^8^

However, only a few studies analyzed the color stability of bleached teeth that were treated with some antioxidant agents.^16^ Most of the antioxidants (natural or synthetic) present pigments in their composition, such as sodium ascorbate (white-yellow crystals), green tea (greenish powder), grape seed extract (brown), α-tocopherol (yellow oil), quercetin (yellowish powder), and quinoa (brown).^8^ In addition, these agents present a low-molecular weight,^8,16^ which allows their capacity to diffuse via the dental substrate. For instance, a recent *in vitro* study demonstrated that the application of sodium ascorbate, immediately after in-office bleaching, significantly reduced the penetration of hydrogen peroxide, but negatively affected the whiteness index achieved by high-concentrated HP.^16^ Thus, it is important to assess whether there is an influence of these substances on post-bleaching color stability. In other words, it is paramount to address whether the recovery of the immediate bond strength would not undesirably affect the esthetic efficacy of bleaching procedures, thereby negatively affecting the patient’s oral rehabilitation.

We found a lack of studies comparing these agents’ antioxidative activity (AA) and, mostly important, their pH’s temporal evolution. Such assessment is important since some agents are directed to be applied longer and could incite changes on the surface of enamel depending on their pH.^9^ Therefore, the objective of this study was to evaluate the pH, the antioxidative activity (AA%), and the impact of different antioxidant agents on the color stability of enamel bleached with 10% HP. The null hypotheses evaluated were that antioxidants (1) would not negatively impact the final esthetic efficacy of a low-concentrated hydrogen peroxide bleaching gel and would not present differences in terms of (2) AA% activity and (3) pH.

## Methodology

### Experimental design

Specimens of enamel bovine blocks (n=80) were randomly allocated into eight experimental groups (n=10/group): GNC (negative control group – no treatment); GPC (positive control group – bleaching only); TOC_10% (bleaching + 10% α-tocopherol); GT_10% (bleaching + 10% green tea extract); GS_5% (bleaching + 5% grape seed extract); SA_10% (bleaching + 10% sodium ascorbate); QUI_10% (bleaching + 10% quinoa extract); and QC_1% (bleaching + 1% quercetin).

Color analyses (ΔE_00_, ΔWI_D_) and pH measurements of the experimental antioxidant’s gels, as well as the percentage of antioxidant activity (AA%) were obtained. Color analyses were performed at four time points: after artificial staining and before bleaching (T_0_ – Baseline), after bleaching (T_1_), after antioxidant application (T_2_), and 14 days after bleaching (T_3_).

A pilot study was conducted to determine the number of specimens for the color analyses (ΔE_00_, ΔWI_D_). Initially, five specimens were evaluated (n=5/group), as previously described. Mean and standard deviations of ∆E_00_ were obtained and statistically analyzed (one-way ANOVA/Tukey’s test), and no differences were found between groups. The mean and standard deviations were used to determine the sample size (G-Power program), with a 5% significance level and 80% power test. The results indicated n=10 as the number of specimens required, in line with previous studies with similar methodologies.^23^ pH measurements and the AA% of the experimental antioxidant’s gels were performed based on previous studies.^5,15^

### Specimen preparation

This *in vitro* study was initiated by selecting 80 bovine teeth that were cleaned and disinfected using a 0.1% thymol solution (Labsynth, Diadema, SP, Brazil). The crowns were then separated from the roots using a diamond disc (KG Sorensen, São Paulo, SP, Brazil) mounted in a high-precision cutter (Isomet 1000, Buehler, Illinois, USA), with a continuous water supply to ensure efficient cooling throughout the process. Enamel blocks, measuring 5×5×3 mm, were subsequently obtained from the central buccal surface of the crown. The dentin surface of these blocks was leveled using a polishing machine (#320) (Arotec, São Paulo, SP, Brazil) to achieve parallelism with the outer enamel surface. Following this procedure, enamel was carefully abraded using silicon carbide sandpaper (#600, 1200, and 2000) and then polished using a polishing cloth and a diamond suspension, containing abrasive particles of 6, 3, 1, and ¼ μm, for 1 min.^23^

### Enamel staining

A thin layer of nail polish was applied covering all faces of the specimens, except for the buccal enamel, which remained exposed. The exposed enamel surface was then immersed in a buffered black tea solution (Dr. Oetker, SP, Brazil, pH=7.0) for 24 h at room temperature with continuous agitation. This solution was prepared by diluting 2 g of black tea in 100 mL of distilled water for 5 min.^23^ Subsequently, the specimens underwent gentle brushing with pumice stone powder to remove non-adherent particles. Afterward, the specimens were stored in artificial saliva (containing 1.5 mM Ca, 0.9 mM P, 150 mM KCl, and 0.1 M Tris, pH 7.0)^24^for 7 d (replaced every 2 d) at 37°C for color stabilization.

### Bleaching procedures and groups distribution

**Figure 1:**
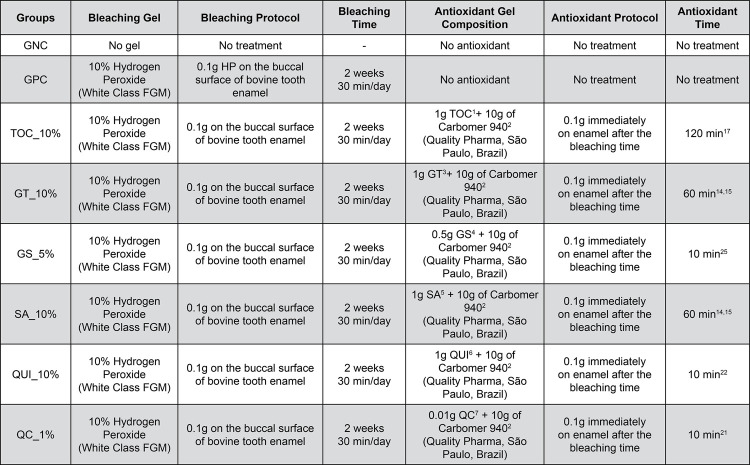
Protocol for the Application of Bleaching and Antioxidant gels on the groups

^1^α-tocopherol 10% extract; ^2^Polyacrylic acid polymer; ^3^Green tea extract 10%; ^4^Grape seed extract 5%; ^5^Sodium ascorbate 10%; ^6^Quinoa extract 10%; ^7^Quercetin 1%

The negative control group (GNC – no treatment) was immersed in artificial saliva^24^ in an incubator throughout the study. The other groups were bleached with a commercial bleaching gel (White Class 10% HP, FGM, Joinville, SC, Brazil). The 10% HP gel was weighed (0.1 g) and applied to the buccal surface of enamel blocks for two consecutive weeks for 30 min/d, following the manufacturer’s recommendations (Figure 1). In the positive control group (GPC), the specimens were only bleached.

### Application of antioxidants

For the other groups (TOC_10%, GT_10%, GS_5%, SA_10%, QUI_10%, and QC_1%), specimens were treated with different antioxidant agents according to the application time recommended by the literature after bleaching (Figure 1). The selection of antioxidant agents evaluated in this study was based on a recent review of the literature on the use of antioxidants in bleached teeth.^8^ All the substances were applied in gel form with the thickener carbomer 940.^14,15,17,21,22,25^ Approximately 0.1 g of TOC_10%, GT_10%, GS_5%, SA_10%, QUI_10%, and QC_1% (Quality Pharma, São Paulo, Brazil) were applied to enamel buccal surface 24 h after bleaching during their respective recommended application times and were rinsed with running distilled water. The antioxidant gels were kept refrigerated, in opaque packaging, and were only opened and removed from the refrigerator at the time of their use, which was immediate. After opening, they had a maximum shelf life of 30 days, as indicated by the manufacturer.

### pH of antioxidant gels

pH was determined using a pH meter coupled to a potentiometer (Orion Research Incorporated, Boston, MA) previously calibrated with pH 4 and 7 standards. The gels were diluted in distilled water in the following proportion: 3 g of gel for 300 mL of distilled water. The pH analyses were conducted under constant agitation at 0, 5, 10, 60, 120, and 180 min, in triplicate, according to the application time of each agent on bovine enamel, as previously described.

### Antioxidant activity percentage (%AA)

To evaluate the AA%, the DPPH free radical assay method was employed.^5,15^ The gels reacted with 0.5mM DPPH stabilized in ethanol solution. The reaction mixture consisted of adding 0.5mL of the sample diluted in ethanol (0.4g sample − 4mL ethanol) to 0.3mL of DPPH solution + 3mL of ethanol. DPPH is reduced when it reacts with an antioxidant component, which can donate hydrogen. The color changes (from deep violet to light yellow) were measured [Absorbance (Abs)] at 517 nm after 45 min of reaction using a spectrophotometer (DU 800, Beckman Coulter, CA, USA). The analysis was performed in triplicate for each substance. The AA% of the substances were determined according to the equation described^26^:AA%=100 − [(Sample Abs − White Abs)/Control Abs × 100].

### Colorimetric evaluation

A digital hand-held spectrophotometer (Easyshade Vita Zahnfabrik, Bad Säckingen, Germany) was used to determine the color coordinates L*(black-white axis), a*(red-green axis), and b*(yellow-blue axis) under controlled lighting, temperature, and atmospheric conditions. To ensure the accurate position of the device tip, the spectrophotometer was fixed to a laboratory clamp, orienting the tip downward. To facilitate contact between the device tip and the specimens, the set was positioned on a white opaque ceramic surface mounted on a lifting platform (Jack lift – Q219, Quimis). This assembly was then situated within a color-matching lightbox, operating in standard daylight mode (GTI Minimatcher Series, GTI Graphic Technology Inc., Newburgh, NY, USA). Subsequently, color measurements were conducted on each specimen from various angles by rotating both the specimen and the ceramic support, all without moving the spectrophotometer.^23^ Color was analyzed at the following time points: before bleaching (T_0_ – Baseline), immediately at the end of bleaching protocol (T_1_), after applying the antioxidant (T_2_), and 14 days after the end of bleaching protocol (T_3_).

The color change (ΔE_00_) was estimated using the CIEDE_2000_ formula: $\Delta E_{00}\left(T_{14}-T_{0}\right)=[{\left(\Delta L^{\prime }/K_{L}S_{L}\right)}^{2}+(\Delta C^{\prime }/{{K_{C}S_{c})}^{2}+{\left(\Delta H^{\prime }/K_{H}S_{H}\right)}^{2}+R{RT}^{*}{\left(\Delta C^{\prime }/K_{C}S_{C}\right)}^{*}\left(\Delta H^{\prime }/K_{H}S_{H}\right)]}^{1/2}$ . The customized Whiteness Index for Dentistry (ΔWI_D_) was estimated following the equation: $WI_{D}=0.511L^{*}-2.324a^{*}-1.100\;b^{*}$ , and the index difference (ΔWI_D_) was determined by T_3_ − T_0_. Color change values (ΔE_00_) adopted for perception (PT) and acceptance (AT) limits (50:50%) were 0.81 (PT) and 1.8 (AT) units. The ΔWI_D_ adopted for PT and AT limits (50:50%) were 0.7 (PT) and 2.6 (AT) units.^27,28^ These Δ values were calculated for three established time intervals: immediately after bleaching (T_1_ – T_0_), after antioxidant application (T_2_ – T_0_), and after 14 days from the procedures (T_3_ – T_0_).

### Statistical analyses

The data obtained were submitted to exploratory analysis using the SPSS 23 software program (SPSS Inc., Chicago, IL, USA). The Shapiro-Wilk and Levene statistical tests determined the normal distribution of the results. Then, one-way ANOVA test with Tukey’s and Dunnett’s tests were applied to compare the control groups (GNC and GPC), with a significance level of 5% for all the variables tested.

## Results

**Figure 2:**
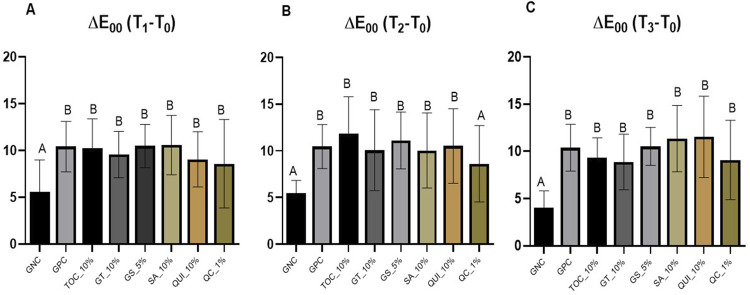
Mean and standard deviation of the color variation (ΔE00) according to the respective treatments and time intervals: (A) immediately after bleaching (T1 – T0), (B) after antioxidant application (T2 – T0), and (C) after 14 days from the procedures (T3 – T0). Different letters indicate statistical differences between groups (p<0.05).

Figure 2 shows the mean and standard deviations of color change (ΔE_00_) according to the respective treatments and time intervals: (A) immediately after bleaching (T_1_ – T_0_); (B) after antioxidant application (T_2_ – T_0_); (C) and after 14 days from the procedures (T_3_ – T_0_). GNC resulted in the least significant color change in bovine specimens, with a statistically significant distinction observed across all evaluated groups and time intervals (p=0.000). However, an exception emerged during the time interval following the application of the antioxidant (T_2_ – T_0_), in which the QC_1% group exhibited no statistical difference from GNC (p>0.05) (Figure 2B). When examining the time interval 14 days after the completion of the bleaching treatment (T_3_ – T_0_) (Figure 2C), the QC_1% group showed a statistical difference from the GNC group (p<0.05), while remaining statistically similar to all other experimental groups evaluated and GPC (p>0.05). This suggests that 14 days after the bleaching treatment, the antioxidant gels evaluated do not significantly impact the color stability of the bleaching treatment and exhibit statistical similarity to the GPC group (p>0.05).

**Figure 3:**
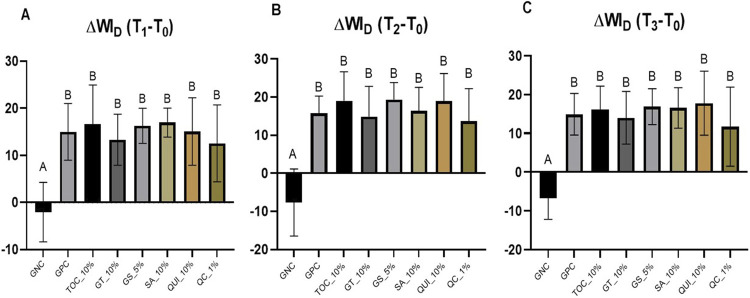
Mean and standard deviation of the Whitening Index for Dentistry (ΔWID), according to the respective treatments and time intervals: (A) immediately after bleaching (T1 – T0), (B) after antioxidant application (T2 – T0), and (C) after 14 days from the procedures (T3 – T0). Different letters indicate statistical differences between groups (p<0.05).

The variation in the ΔWI_D_ was statistically different only for GNC in comparison to the other groups, regardless of the evaluated times (p<0.05) (Figure 3 A-C). This implies that the various antioxidants evaluated did not influence the color stability of enamel treated with low-concentration HP bleaching.

**Figure 4:**
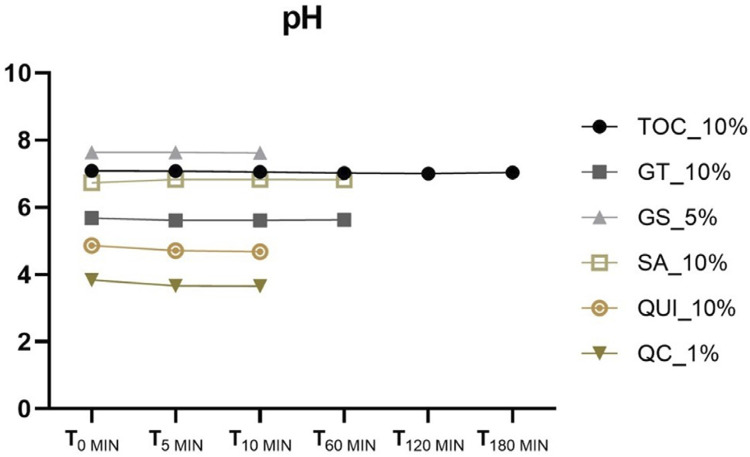
Mean of the pH values of the antioxidant gels according to the evaluation times.

The QC_1% antioxidant gel displayed the most acidic pH, with values ranging from 3.64 to 3.67, indicating its potential for erosive effects. In contrast, the GS_5% solution exhibited the least acidic pH and was the most alkaline among the gels examined (Table 1). In terms of pH changes over the assessment period, a consistent pattern of gel stability was evident (Figure 4).

**Table 1:** Mean and standard deviation of the pH values of the studied antioxidant gels. Different letters indicate statistical difference (p<0.05).

Antioxidant Gels	pH	*p* value
TOC_10%	7.07 (0.02)^A^	0.001
GT_10%	5.65 (0.03)^B^	
GS_5%	7.64 (0.02)^C^	
SA_10%	6.83(0.01)^D^	
QUI_10%	4.75 (0.09)^E^	
QC_1%	3.64 (0.01)^F^	

**Figure 5:**
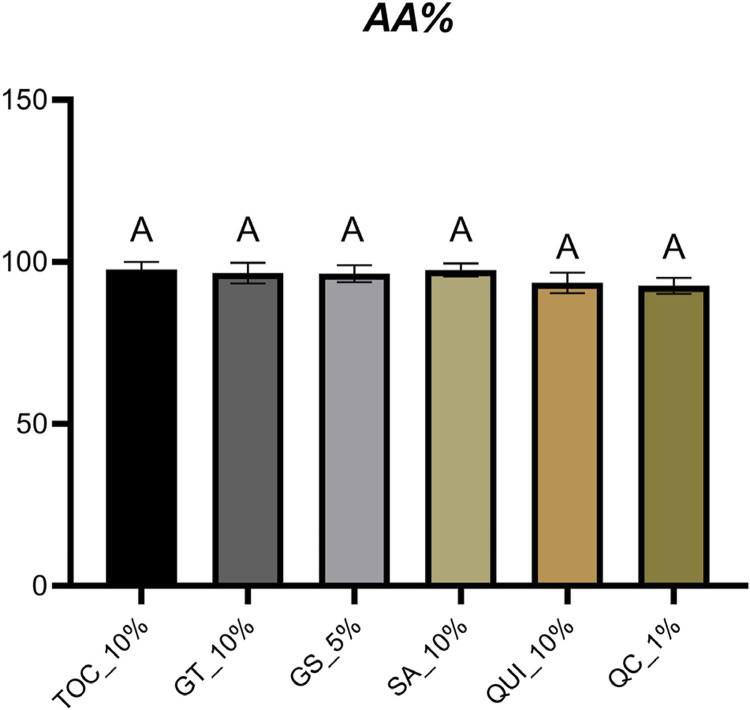
Mean and standard deviation of the antioxidant activity (AA%) values of the antioxidant gels. Same letters indicate no statistical difference (p>0.05).

All antioxidant gels under examination exhibited a notably high level of antioxidant activity (AA%), with values ranging from 92.61±2.4 (QC_1%) to 97.66±2.33 (TOC_10%) with no statistically significant distinctions among them (Figure 5).

## Discussion

Tooth bleaching, without further antioxidant application, achieved satisfactory mean ΔE_00_ and ΔWI_D_ in this study. The following application of all the AA into the enamel surface did not significantly affect those esthetic parameters except for the QC_1%, which presented significantly lower ΔE_00_ than all the other groups evaluated, but only at T_2_ (immediately after bleaching). However, all the bleached groups did not exhibit significant differences after 14 days in artificial saliva, thereby showing that the final esthetic efficacy of bleaching with 10% hydrogen peroxide was not affected by any of the tested antioxidants application. Therefore, the first null hypothesis was accepted. We highlight that both color evaluation methods (ΔE_00_ and ΔWI_D_) were selected for this analysis, as they represent the most current indices used for color assessment in dentistry.^27,28^

We found scarce evidence of the antioxidant effect on color outcomes from tooth bleaching.^8,16^ However, several authors have already demonstrated the positive effect of sodium ascorbate, α-tocopherol, grape seed extract, and green tea on the recovery of enamel bond strength following treatment of enamel with peroxides.^14-21^ In this context, our investigation could answer this open question, addressing that these widely investigated antioxidants would not downgrade the highly acceptable esthetic outcomes reached by at-home bleaching.

Nevertheless, we highlight that 1% quercetin showed similar results to GNC in terms of color change (ΔE_00_) immediately after bleaching (T_2_). However, at T_3_, ΔE_00_ and none of the ΔWI_D_ results indicated significant differences among QC_1%, GPC, and all the antioxidants’ groups. The final ΔWI_D_ showed mean differences that were higher than the perceptibility and acceptability thresholds.^28^ A feasible explanation for the ΔE_00_ result could be the color (yellowish powder) and low pH of QC_1% during the application, which was confirmed in this study. The more acidic pH can alter the dental enamel microstructure, allowing the gel to penetrate deeper and, due to its color, affecting the reflection pattern and altering color analysis.^29^ Another fact is that the standard deviation for this group was high, and this large variation possibly favored the similarity with the GNC group. Notably, quercetin has been evaluated by Shamsedin, et al.^21^(2017), who pointed out that this flavonoid could reverse the post-bleaching bond strength of orthodontic brackets under the same QC concentration (1%), which was confirmed by the high AA activity herein evaluated. However, the authors did not state any concerns about the color stability of this acidic compound. Based on our findings, further studies are paramount to determine the ideal quercetin solution concentration and pH to be used after tooth bleaching.

Another flavonoid with scarce evidence in the literature is quinoa. Previous studies on quinoa mainly focus on its dietary benefits, nutritional value, and disease prevention potential.^30^ However, the scarcity of studies in Dentistry limits our understanding of its possible applications in this field, despite its recognized antioxidant potential. Regardless of its brownish color and acidic pH, which was higher than that observed for quercetin, quinoa did not affect the tooth bleaching efficacy. The AA% analysis confirmed that quinoa presents a noticeably high antioxidant activity. None of the antioxidants studied presented significant differences among each other, accepting the second null hypothesis. According to previous research,^31^ the interaction of an antioxidant with the DPPH radical depends on its molecular structural form, that is, the number of reduced DPPH molecules is directly linked to the amount of hydroxyl groups (OH^−^) available since it is a redox in which the antioxidant undergoes oxidation (loss of electrons) and the DPPH radical undergoes reduction (gain of electrons). In this direction, it is expected that future studies will show that the natural compound quinoa, which is widely used in other areas and food industry,^30,32^ is not only able to uphold enamel color change but also to recover the bond strength.

An interesting behavior was observed in the present study regarding the ΔE_00_ values of the negative control group, which exceeded the perceptibility threshold^27,28^, such as the other experimental groups. However, when examining the ΔWI_D_ values, a different pattern is evident compared to the other groups, with the means showing negative values. This indicates that the samples in this group showed a darker appearance, which may be justified by the lack of whitening treatment and its stabilization via interaction with artificial saliva. There was no color maintenance of the specimens in a positive direction, as observed.^33^

Another crucial factor for clinical applicability analysis is checking the pH of the antioxidant. The critical pH for demineralization in enamel is 5.5 and in dentin is 6.5. Thus, applying substances to the dental structure with pH values below this threshold could lead to greater demineralization, porosity, and consequently greater dental sensitivity to the patient.^29^ In this sense, the third and final hypothesis was rejected, as there was a statistical difference in the pH of the antioxidant gels evaluated in this study. Due to their high acidity, QUI_10% and QC_1% should be used with caution as they can lead to enamel demineralization, turning dental enamel more susceptible to extrinsic pigmentation and sensitivity.^34^ On the other hand, given that both were applied for only 10 min, the lingering question of whether there might be adverse implications for an erosive process persists, considering this relatively brief duration.

Among all these substances, ascorbic acid is by far the most extensively studied.^35^ It is one of the salts derived from vitamin C. Tocopherol (TOC), derived from vitamin E, is mentioned less frequently in the literature. Although this study does not have the main objective of evaluating the increase in bond strength after the use of antioxidants on bleached enamel, both flavonoids mentioned above are cited in studies^17^ as effective substances for reversing bond strength when applied after the bleaching protocol. These antioxidants have more neutral intrinsic colors (white to yellow and pale yellow, respectively). Therefore, when using them on the surface of the bleached enamel, it was expected that the color stability after bleaching would not be affected, a fact that was confirmed via color evaluations.

However, a recent study^16^ investigated the effect of applying 10% SA gel for 10 min after in-office bleaching with 35% HP on color stability and showed that 10% SA interfered with the bleaching process. The authors attributed this interference to the capacity of SA to reduce the diffusion of HP via enamel and dentin, thus compromising the effectiveness of bleaching. Despite this interference, significant bleaching effects were observed in both groups (with and without SA application) since the difference between the group means for ΔE_00_ did not exceed the perceptibility and acceptability values of 50:50%. This suggests that, although some significant differences were observed between the groups, the research results cannot be fully interpreted without comparing perceptibility and acceptability thresholds. However, this relationship was not observed for ΔWI_D_, as the difference between the means of this variable exceeded the perceptibility and acceptability.^28^ Despite the discrepancies between the results of this study and the previous study^16^ regarding ΔWI_D_, it is important to consider the differences in the bleaching protocol and the dental substrate used. The authors of the previous used enamel from human molars, and there is no detailed information on the composition of the 10% SA gel, or the amount applied to the enamel surface. Therefore, further *in vitro* studies should be conducted considering all these variables to achieve a comprehensive understanding of their impact on whitening efficacy.

Contrary to expectations, both GT and GS, as they have darker intrinsic tones (greenish and brownish, respectively), raised the assumption that they could affect the color stability of the enamel. However, like other antioxidants, these gels also did not cause changes in the color stability of the enamel subjected to bleaching with 10%HP. In this context, although antioxidant agents are low-weight molecules capable of penetrating the enamel pores after bleaching,^8,16^ it appears that the time used for applying gels to the enamel surface is insufficient for the molecules to reach the dentin and cause color changes. Consequently, this favors the use of these substances to increase the bond strength of the bleached enamel without compromising the color stability in the bleaching treatment.

Researchers^14,15^ have reported that the bond strength values were higher than the control group (only bleached) when 10% GT was applied after enamel bleaching for 60 minutes. The same was not observed at 15 and 30-minute intervals or even at concentrations of 20% and 30%. According to De-Carvalho, et al.^15^(2016), it is speculated that the antioxidant in gel form with these higher concentrations produces more substance powder. This could lead to greater material deposition on the enamel if not completely removed by washing and conditioning, resulting in lower bond strength values. However, other researchers^36^ used it for 10 min and obtained a significant increase in bond strength. Lack of standardization occurred with all antioxidant substances involved.

Furthermore, the bleaching treatment performed was home bleaching with 10% hydrogen peroxide (HP), in contact with dental enamel for 30 min/d for 14 d. However, there are too many variations in the bleaching protocols mentioned in the literature associated with antioxidants. For example, Vidhya, et al.^25^(2011) used 38% HP for 10 min; Khamverdi, et al.^36^(2013), 40% HP for 10 min; Sasaki, et al.^17^(2009), 10% Carbamide Peroxide (CP) for 2 h/14 d and Berger, et al.^14^(2013), 10% CP for 8 h/14 d. This protocol divergence complicates proper applicability and should be carefully considered in future studies related to the topic. However, it is worth noting that, clinically, the use of these antioxidants is still unfeasible due to the various application methods addressed in the literature. Moreover, some of these antioxidants hold a very long-acting time, such as α-tocopherol (TOC), which requires about 120 min in contact with the dental structure for its efficacy.^17^ In addition, these gels are usually prepared in pharmacies and, therefore, present a short shelf life.

Considering the limitations of this study, it is essential that additional *in vitro* and, subsequently, *in situ* studies are conducted. These future studies should establish standards for the time and concentration of antioxidants and the bleaching protocol, ensuring the safety of using these flavonoids in human dental enamel. One might say that the use of black tea to stain specimens before bleaching procedures could be a limitation since hydrogen peroxide-based products act by breaking down the intrinsic pigments in dentin. However, artificial staining with black tea has been widely used in various whitening studies^7,23,37^ to standardize color parameters. In this context, we highlight that all specimens were subjected to the same protocol and then randomized into groups with no differences in the initial mean values of the L*, a*, and b* coordinates. Additionally, the evaluation of the enamel surface must be conducted considering that some of the antioxidants evaluated show a low pH, which could induce demineralization on the surface. This is essential to obtain more accurate conclusions, increasing the clinical application of these substances.

## Conclusions

The antioxidant gels studied showed satisfactory AA% and demonstrated that they did not alter the color stability of enamel bleached with 10% HP. However, quinoa and quercetin exhibited low pH levels and their use must be cautious as they can cause structural changes in tooth enamel.
